# Nest Entrance Architecture and the Regulation of Foraging Activity in Desert Harvester Ants

**DOI:** 10.1002/ece3.72122

**Published:** 2025-09-06

**Authors:** Deborah M. Gordon

**Affiliations:** ^1^ Department of Biology Stanford University Stanford California USA

## Abstract

The architecture of an ant colony's nest entrance modulates the regulation of activity in and out of the nest. This study considers how the architecture of nests of the desert harvester ant *Pogonomyrex barbatus* facilitates the regulation of foraging activity in an arid environment. Colonies must spend water, in water lost to evaporation when outside the nest, to obtain food and water. Previous work shows that encounters in the chamber just inside the entrance function as the valve to manage this tradeoff by regulating whether foragers decide to leave the nest on another trip. Here both complete and partial excavations, and observations inside active nests, were made in a long‐term study population in New Mexico, US. Both the overall nest architecture and the set of chambers around the nest entrance are structured as a minimum spanning tree with the entrance chamber as hub. The entrance chamber is surrounded by 1–6 adjacent chambers not linked to each other, with 2–3 tunnels that lead to strings of widely spaced chambers descending 1–2 m. Observations with a videoscope inside active nests show that exterior workers of different tasks move up to the entrance chamber each day during foraging activity and descend below it afterwards, and that interior workers quickly carry food down from the entrance chamber to the deeper nest. Larger, older colonies have more nest entrances with tunnels leading to a single entrance chamber than younger, smaller colonies; this may reduce variability in encounter rates. The nest entrance architecture facilitates rapid adjustment of activity outside the nest. However, in current, deepening drought conditions, it is susceptible to damage when the upper clay layer of the calichi soil dries out, disrupting encounters in the entrance chamber and inhibiting the colony's capacity to manage water loss.

## Graphical abstract

Colonies of desert seed‐eating ants lose water to evaporation when out foraging and get water from the seeds they eat. They regulate foraging using encounters in the chamber just inside the entrance. The entrance chamber is a hub that channels ants inside the nest directly to the entrance. Drought induced by climate change dries the soil, which damages this structure and makes it more difficult for the colony to manage water loss.

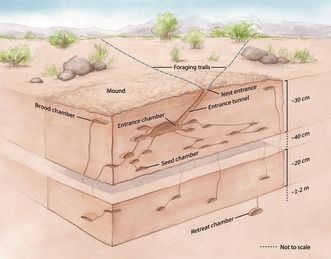

The evolution of collective behavior, like any phenotypic trait, depends on how it functions in relation to ecological conditions (Gordon 2023). Ant nest architecture provides beautiful examples of the outcomes of collective behavior, as ant colonies operate without central control to build, maintain, and use their nests. Recent advances in the study of nest architecture have led to innovations in measuring nest structure (e.g., Tschinkel 2021) and in evaluating nest topology (e.g., Pinter‐Wollman 2015a), and show how simple interactions are used to construct nests (e.g., Perna et al. 2017). This work has revealed enormous diversity of nest structure among ants (Buhl et al. 2006; Miller et al. 2022; Tschinkel 2015, 2021), opening the way to learn how this diversity reflects differences in ecological conditions.

Just as the structure of a building influences the movement of people in it (Helbing et al. 2005; Nisinari et al. 2006), nest structure determines how ants move around (e.g., Franks and Tofts 1994; Dussutour et al. 2004; Pinter‐Wollman et al. 2012). Since movement patterns determine encounter rates (Gordon 2020), nest structure influences when and how often ants interact (Burd et al. 2010; Davidson and Gordon 2017; Pinter‐Wollman 2015a; Pinter‐Wollman et al. 2013, 2018; Tschinkel 2015; Vaes et al. 2020).

A useful way to characterize ant nest structure is to visualize it as a network, with chambers as nodes and tunnels as edges (e.g., Buhl, Gautrais, Deneubourg, and Theraulaz 2004; Buhl, Gautrais, Sole, et al. 2004; Buhl et al. 2006; Pinter‐Wollman 2015a). The connectivity of the nest network is the extent to which nodes, or chambers, are linked to each other by tunnels. As in human transport networks (Marin et al. 2022) or data networks (e.g., Fenci et al. 2011), connectivity determines the flow of ants around the network, (Pinter‐Wollman 2015a; Miller et al. 2022; Vaes et al. 2020). Ant species differ greatly in nest connectivity (Miller et al. 2022), which is strongly associated with ecological conditions (O'Fallon et al. 2023).

The connectivity of the nest entrance and adjacent chambers sets up the patterns of interaction that regulate the flow of ants in and out of the nest. (Gordon 1996, 2010, 2019; Pinter‐Wollman 2015b; Pinter‐Wollman et al. 2018; Lehue and Detrain 2019). The regulation of activity outside the nest responds to ecological conditions, such as how frequent and severe are the risks of danger outside, and the costs of missing opportunities by staying inside (Gordon 2019, 2023). Differences among species in nest architecture can determine how quickly the colony reacts to changes outside the nest, and how resilient is the structure to restore function after a catastrophe (O'Fallon et al. 2023; Sankovitz and Purcell 2021).

Here I consider how the architecture of the nest, particularly near the nest entrance, of the red harvester ant *Pogonomyrmex barbatus*, is related to the ecology of this seed‐eating species in the desert. In arid environments, ants lose water to evaporation when outside the nest (Feener and Lighton 1991). Seed‐eating ants obtain water from metabolizing the fats in the seeds (Lighton and Feener Jr. 1989). Thus desert seed‐eating ants must spend water lost while foraging, to obtain water and food from the seeds the foragers collect. Colonies of *P. barbatus* manage this tradeoff by regulating foraging activity in response to changes in food availability and humidity (Pagliara et al. 2018). Foragers make many trips to search for and collect seeds. A forager's decision whether to leave the nest on its next trip is based on its rate of encounter, in brief antennal contacts, in the entrance chamber just inside the nest entrance, with foragers returning with food (Pinter‐Wollman et al. 2013; Davidson et al. 2016). In an antennal contact, one ant assesses the cuticular hydrocarbon profile of the other (Greene and Gordon 2003). Feedback based on forager return rate provides a cue to food availability, because each forager searches until it finds food (Beverly et al. 2009); the more food is available, the more quickly it returns. A forager's decision whether to leave on its next trip also depends on the desiccation it experienced on previous trips, and thus on the risk of water loss (Pagliara et al. 2018; Nova et al. 2022).

The observations reported here were made at or near a long‐term study site near Rodeo, New Mexico, where a population of *P. barbatus* colonies, about 300 colonies per year, has been monitored and censused since 1988 (Sundaram et al. 2022), so that the ages of all colonies are known. I first review previous work on the overall structure of a nest of a *P. barbatus* colony, adding observations from complete excavations of 30 nests. Next, I outline observations from excavations of the nest entrances and adjacent chambers of a further 19 nests, to learn how the entrance chamber and adjacent chambers are connected, and to consider how this architecture influences the flow of ants in and out of the nest. High connectivity, with the chambers adjacent to the nest entrance linked to each other and to chambers further down, would provide many possible routes for ants to access and disperse from the entrance chamber. By contrast, low connectivity among adjacent chambers, making most of them dead ends, would act to funnel ants from lower in the nest directly to and from the entrance chamber and outside.

I then consider how the nest entrance structure changes as a colony grows older and larger, and how this is related to the regulation of foraging activity. A colony grows from about 2000 workers when it is 2 years old to a stable size of 10,000 workers when it is reproductively mature at 5 years old (Gordon 1992, 1995) and for the rest of its 20–30 year lifespan, usually in the same nest (Ingram et al. 2013; Sundaram et al. 2022). Returning foragers travel through an entrance tunnel to reach the entrance chamber. The more directly returning foragers encounter outgoing ones, the more precisely the encounter rate reflects the true rate of forager return, the cue to food availability. As a colony grows, it could make the entrance tunnel wider to accommodate more ants entering and leaving the nest. However, the wider the tunnel, the more opportunity for eddies and turbulent flow. Alternatively, the colony could add more narrow tunnels and use more entrances out of a common chamber (Lehue and Detrain 2019). Narrow tunnels promote head‐on encounters in ants (Dussutour et al. 2004; Dussutour et al. 2009; Fourcassie et al. 2010; Couzin and Franks 2003), just as narrow sidewalks do for human pedestrians (Nisinari et al. 2006), and so would streamline encounters with returning foragers.

Next I bring together observations of nest entrance structure and of ants inside active nests, along with results of previous work, to surmise the daily pattern of movement of exterior workers in and out of the nest. The effects of nest structure on ant movement have been studied mostly in laboratory colonies (e.g., Monaenkova et al. 2015; Buhl, Gautrais, Deneubourg, and Theraulaz 2004; Perna et al. 2017; Vaes et al. 2020), because excavation of an ant nest destroys it, though it is possible to observe the activity of ants in partially excavated nests (e.g., Tschinkel and Hanley 2015). Here I monitored the flow of ants into and out of the entrance chamber in active nests in the field, using partial excavations and observations with a videoscope inside nests.

In the last section I describe the impact on nest entrance structure of recent drought conditions. The long‐term study began in the late 1980's when rainfall was high; the years 1980 to 1998 were the wettest, while 2000–2018 approach the driest, in about 1100 years (Williams et al. 2020). Temperatures continue to rise while rainfall is declining. The ongoing drought has intensified competition among neighboring colonies for foraging area (Sundaram et al. 2022), because reduced rainfall diminishes the supply of the seeds that the ants eat.

High temperatures and low humidity make the nest entrance vulnerable to cracking and to wind erosion. Below about 10 cm, the calcrete or calichi soil (Soil Survey USDA 2024, Buol et al. 2011) is too hard to absorb water from rainfall. The nest entrance is built in the top clay layer of the soil, where rainfall is absorbed and where water tends to move laterally as it is pulled from wetter areas to dry ones by plants (Scott and Biederman 2019). This clay layer swells when wet, and shrinks when dry (Bretz and Horberg 1949), with drastic effects observed in recent years.

## 1 Nest Structure

### 1.1 Overall Nest Architecture

Since 1988 about 30 nests have been excavated in vertical slices, including those of 6 colonies of known age and an additional 4 estimated to be mature (≥ 5 years old) described in Gordon (1992), and other nests excavated to collect queenright colonies to bring back to the lab. In all of these full nest excavations, a trench was dug beside the nest, at times with the help of a backhoe, and then vertical cuts were made in the rock from the side of the trench with pickaxes, rock hammers, and trowels, to dig a hole in the rock about 2.5 m deep and 3–4 m wide.

The nest mound of a mature colony is about a meter wide. The study population has two dependent lineages, and the mean (SD) area of the nest mounds is 2.82 m^2^ (1.69) in J1 and 2.27 m^2^ (1.66) in J2 (Gordon et al. 2013). The surface of the mound is covered by small pebbles that the ants collect and then imbue with colony‐specific cuticular hydrocarbons that help to guide foragers back to the nest (Sturgis et al. 2011). The pebbles may also help to trap moisture on the mound surface.

**FIGURE 1:**
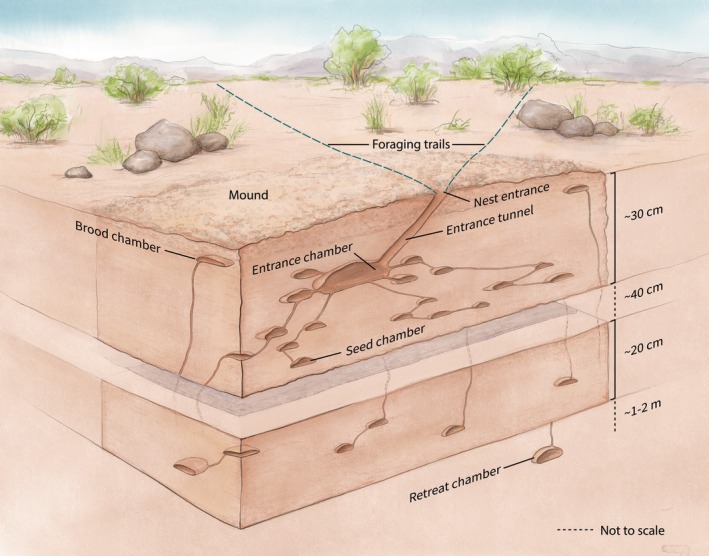
A nest of *Pogonomyrmex barbatus* . Illustration by Semay Johnston. A photo of a cross section of an excavated nest is shown in Figure 2A.

**FIGURE 2:**
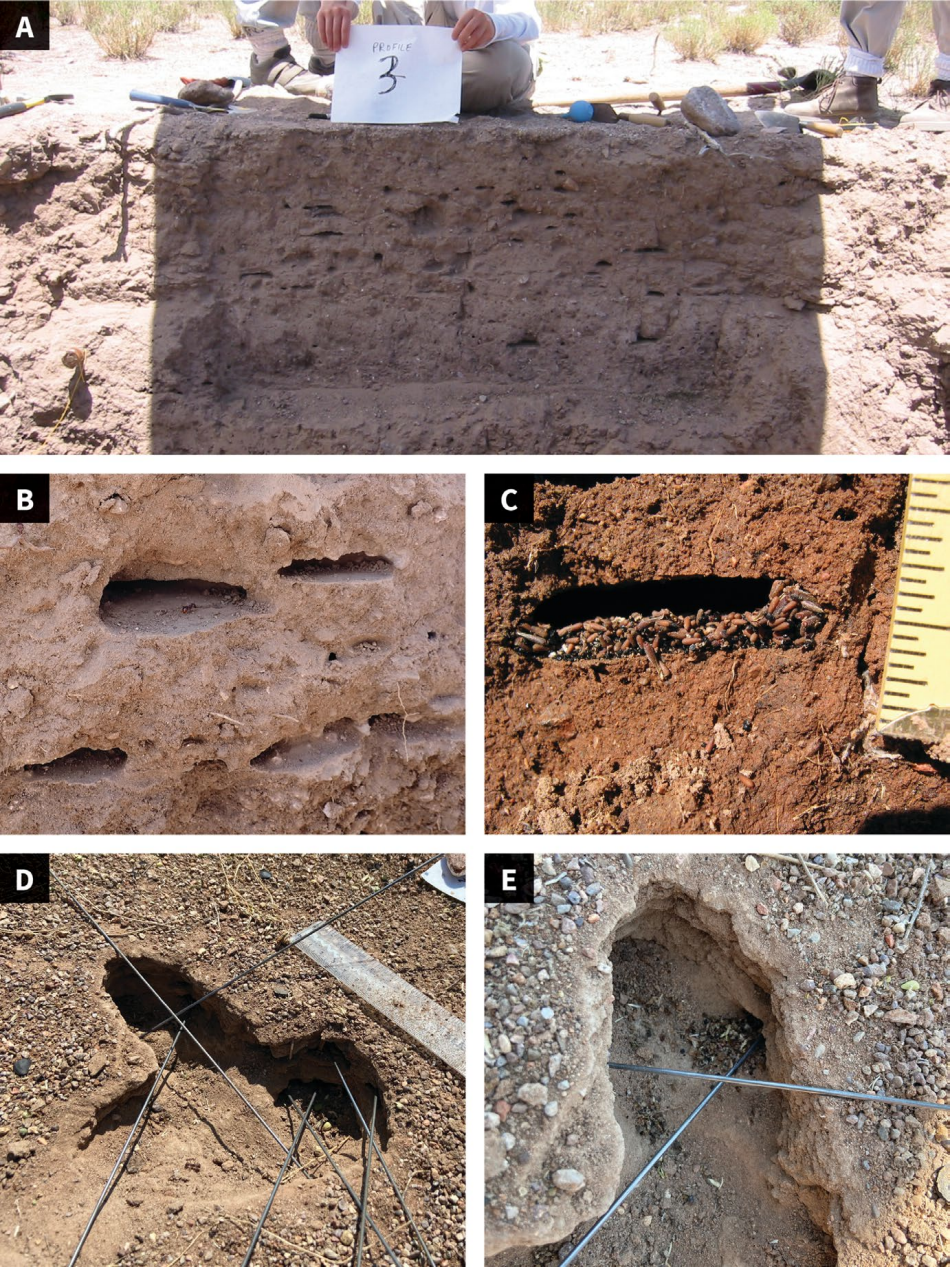
(A) Cross section of an excavated nest of a mature colony. The piece of paper held at the surface is 28 cm wide. Aug 2003, photo by Sam Crow. (B) Chambers in an excavated nest, lined with a packed adobe surface. Aug 2024, photo by the author. (C) Seed chamber. Intervals between lines on the ruler are 0.3 cm. Aug 2004, photo by Miko Tsukimoto. (D) Entrance chamber with wires in all tunnels leading out of the chamber. Aug 27, 2020, (Figure 5C), photo by Sophie Eisenberg‐Edidin. (E) Entrance chamber with wires in tunnels to adjacent chambers. On the left, a pile of rubbish to be taken out of the nest; on the right, a pile of seeds to be taken deeper into the nest. This image was enhanced for visual clarity to remove a third wire using generative AI. Aug 22, 2020, photo by Sam Crow.

As reported for other species of *Pogonomyrmex* with large colonies, including *P. badius* (Tschinkel 1999, 2004) and *P. occidentalis* (Lavigne 1969; Halfen and Hasiotis 2010), the underground nest is a cone‐shaped mass of chambers. As in *P. occidentalis* (Lavigne 1969; Halfen and Hasiotis 2010), the vertical axis of the main mass of chambers is about as long as the mound is wide (Figures 1 and 2A).

The cluster of dense chambers surrounding the nest entrance can extend down 20–30 cm below the surface, in a large mature colony 5 years or older (Gordon 1992). There are more sparsely distributed chambers down to about 1 m below the surface. These lower chambers are more likely to be under the center of the mound than at its edges. Extending from these lower chambers are long chains of chambers and tunnels that go off to the side, or down, to chambers linked by one other tunnel to another chamber. These chains of tunnels and chambers are not connected to a central tunnel.

While most chambers surround the nest entrance in the middle of the mound, there are also peripheral chambers at the edges of the mound just under the surface where brood is brought to warm in late morning (Figures 1 and 2A). The tunnels from these chambers tend to lead directly down to link with chambers below, not horizontally to the nest entrance.

In one excavation in 2003, 4 vertical slices about 10 cm apart were photographed (one is shown in Figure 2A). Chamber size was measured from these photos using GIMP software to measure the number of pixels in a line on the photo across the width of each chamber. Chamber width ranged from 0.6 to 7.8 cm, with a mean (SD) width of 4.5 (5.0), *n* = 78. No chambers appeared in successive photos, as the distance between vertical slices was larger than the width of the chambers.

At the bottom of the nest, there is a long tunnel that extends as much as 1 m below the other chambers, to a depth of 2 m or more in a mature colony (Figure 1, ‘retreat chamber’), as observed by Lavigne in *P. occidentalis* (1969), and in *P. barbatus* by McCook (1879), who reports that one large nest had a tunnel extending to a chamber 5 m below the surface. During a nest excavation, as hard calichi soil is assaulted with pickaxes and trowels, the ants respond by carrying brood and seeds out of exposed chambers into tunnels toward chambers with no other exit. The pupae and larger larvae are found packed into isolated chambers at the bottom or sides of the excavated pit, with callows (light‐colored workers within 2–3 days of eclosion as adults from pupae) and other workers. The queen is with the youngest brood, eggs and tiny larvae, as well as callows and workers. I do not know how the lower chambers are used in ordinary conditions.

**FIGURE 3:**
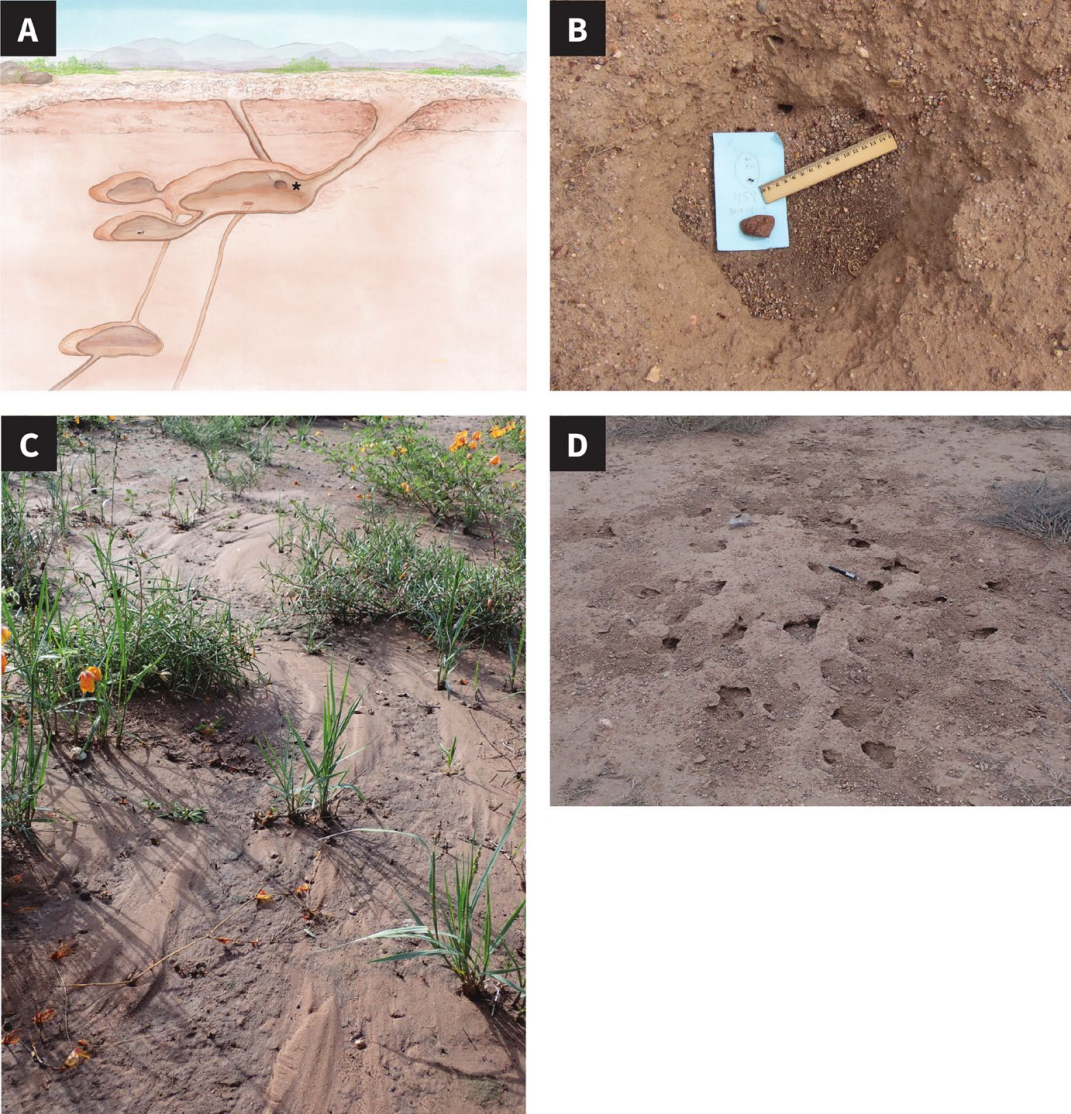
(A) Two nest entrances lead to entrance chamber. The star indicates the wall between the two entrances that may help to reduce turbulence in the flow of ants in and out of the entrance chamber. Illustration by Semay Johnston. (B) Nest entrance collapsed from wind erosion in dry conditions, Aug 2019. (C) Mud flow from flooding, Aug 2022. (D) Nest exposed, Aug 2023. All photos by Sam Crow.

It seems likely that the long tunnels to isolated chambers deep in the nest are used to retreat when the ground is flooded during heavy rain, and water gets into the nest entrance and linked chambers, forcing the ants to go lower. After flooding, some colonies remain inactive under layers of mud (Figure 3C) until there are a few days of dry weather. Eventually, ants emerge carrying out wet soil from a newly dug nest entrance.

As in *P. occidentalis* (Lavigne 1969) and in many other ant genera (Tschinkel 2021; O'Fallon et al. 2023), the nests of larger colonies generally have more chambers. The depth of the nest, excluding the escape tunnel and chamber, is about the same as the diameter of the mound; it expands to about 1 meter as the colony grows older.

The floors of all chambers are remarkably flat, and the ceilings are curved (Figure 2B). Each chamber is entirely lined with an adobe‐like surface, made by applying moist soil to the walls and floor of the chamber and packing it down until smooth. McCook (1879) noted that chambers of excavated nests of *P. barbatus* in Texas were lined with soil from the surface, as did Halfen and Hasiotis (2010) for *P. occidentalis* . There are two apparent functions of this dense surface: to hold in moisture and to provide structural support to the nest. In laboratory colonies, ants line the walls and ceilings of their plastic nest boxes with moist soil if soil is provided. The flat floors may be packed down at least in part by ant movement, but the ceilings and walls must be generated by other activity, as observations with a videoscope, described below, suggest that ants walk only on the floor. Further work is needed to determine the function of the hard lining of the tunnels and chambers.

Chambers with stored seeds are deep in the nest without many connections to other chambers (Figure 2C), as in other seed‐eating ant species including *P. badius* (Kwapich and Tschinkel 2013, 2016). Often different seed species are placed in distinct layers in the seed chamber. Excavations in the late summer reveal chambers filled with seeds that are available only later in the fall, indicating that seeds can be stored for at least 9–12 months.

## 2 The Entrance Chamber

### 2.1 From the Outside to the Entrance Chamber

**FIGURE 4:**
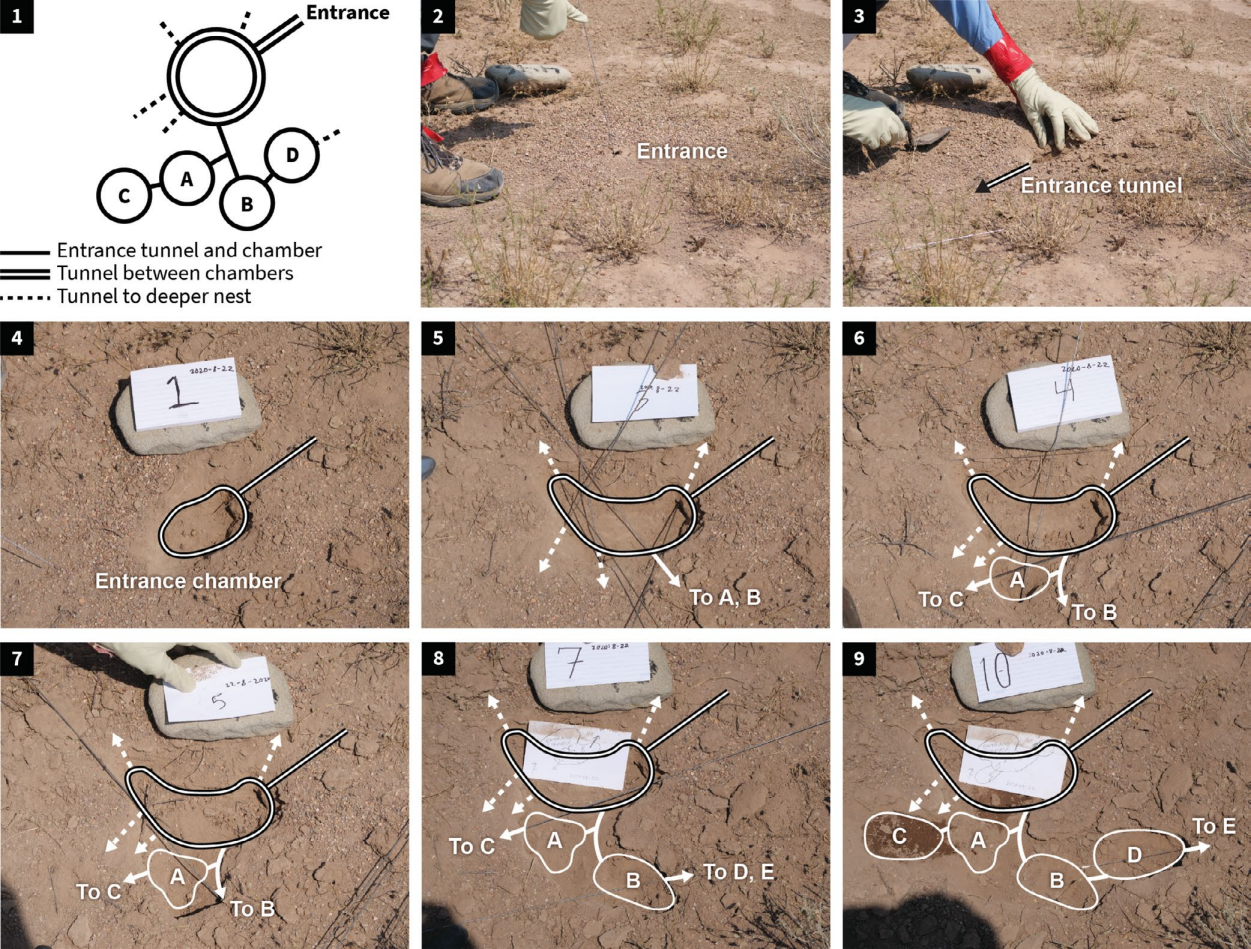
Excavation of the entrance chamber and adjacent chambers. Photos by Sam Crow and Sophie Eisenberg‐Edidin, Aug 222,020. The paper label is 12.7 cm wide. (1) Diagram of excavated chambers (Figure 5B) (2); Wire in nest entrance before excavation; (3) Entrance tunnel from nest entrance to entrance chamber; (4) Entrance chamber excavated; (5) Wires in all tunnels leading out of entrance chamber (as in Figure 2D);(6) Adjacent chamber (A) excavated; (7) Tunnel to adjacent chamber (B) excavated; (8) Adjacent chambers (A, B) excavated; (9) All adjacent chambers (C, A, B, D) excavated. The soil in C is darker because it was recently excavated and still humid.

To examine the structure of the nest entrance and adjacent chambers, I excavated nest entrances in 9 colonies in 2010, 4 in 2012, 3 in 2013, and 3 in 2020, for a total of 19 colonies. I lifted the soil in a horizontal layer with a trowel and spoon, making it possible to trace all links among chambers. Beginning from the nest entrance, I removed the soil around the entrance tunnel, then exposed the entrance chamber and all chambers adjacent to it (Figure 4). Because the chambers are lined with a layer of hard soil, it is possible to remove all looser soil with a spoon, to reveal the outlines of a chamber. I put a wire flag into each tunnel to trace the links between chambers as the tunnels were removed (Figures 4 and 2D). After these partial excavations, most colonies fully repaired the nest entrance in 1–2 weeks.

All excavations of nest entrances were done at the end of the morning activity period. Colony age, which is associated with colony size (Gordon 1992), was known from the annual census of the population (Sundaram et al. 2022) for the 3 colonies from the study population that were excavated in 2013, and estimated for the other 16, which were near but not on the study site, by comparing nest mound size with those of colonies of known age on the study site. Nest mound size varies greatly from year to year in all colonies (Wagner et al. 2004; Gordon et al. 2013), but in a given year, colonies of the same age tend to have about the same size mounds. The 19 colonies were known or estimated to include 3 colonies of age 1–2 years, 3 aged 3 years, 1 of age 4 years, and 12 of age ≥ 5 years.

Entrances to the nest at the mound surface vary broadly in shape and width, from small holes about 1 cm wide, to large flat openings about 2–3 cm wide, with the mound surface overhanging a runway that narrows into the entrance tunnel.

The entrance tunnels from the nest entrance at the surface down to the entrance chamber are narrower than the nest entrance itself, about 1–2 cm wide (e.g., mean (SD) 1.4 (0.4) cm, *n* = 9 measured in 2010), enough to hold up to 25 ants at any cross‐section. This corresponds to the highest rates, about 25–30 ants per second, at which foragers leave the nest on the trail (Gordon et al. 2011; Prabhakar et al. 2012). The entrance tunnels are 5–10 (mean (SD) 7.9 (2.1) cm, same *n* = 9 measured in 2010) cm long, with the same width along the tunnel, with a downward slope of about 2 cm down for every 3 cm in horizontal distance. These 1–2 cm tunnels from the outer entrance to the entrance chamber are much wider than the tunnels between chambers, which usually are less than 1 cm wide, and are difficult to measure as they tend to collapse when excavated.

### 2.2 From the Entrance Chamber to the Rest of the Nest

**FIGURE 5:**
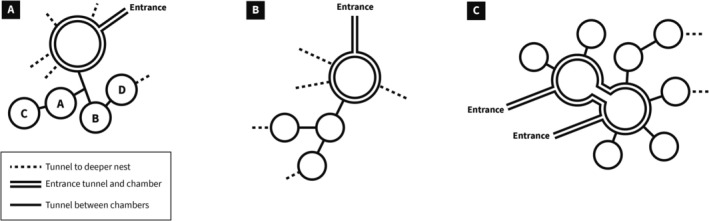
Diagrams of entrance chamber and adjacent chambers (A) Young colony < 5 years, 8‐22‐2020 (excavation illustrated in Figure 4); (B) Old colony ≥ 5 years, 8‐24‐2020, (C) Old colony > 5 years, 8‐27‐2020. Dotted line, tunnel to deeper nest; double line, entrance tunnel and chamber; solid line, tunnel between chambers.

In the 19 nests with entrance chambers excavated horizontally, the chambers varied in size and shape, but the topology of the entrance chamber and adjacent chambers was the same: the entrance chamber is connected by small tunnels to several other adjacent chambers. The chambers adjacent to the entrance chamber are not linked to each other, creating a minimum spanning tree formation (Figure 5). These adjacent chambers often contain piles of seeds or rubbish such as seed husks to be taken out (Figure 2E). The tunnels from the entrance chamber to adjacent chambers were 0.5–1 cm wide, similar to other tunnels inside the nest apart from the entrance tunnel (Figure 1). Adjacent chambers were usually along the sides of the entrance chamber furthest from the exit to the outside via the entrance tunnel.

The entrance chamber is generally larger than any chambers seen elsewhere in the nest. Entrance chambers from 9 nests measured in 2010 had a mean (SD) longest axis of 7.3 (0.9) cm and a perpendicular axis of 3.7 (1.3) cm. Entrance chambers from 3 nests each in 2012 and 2020, measured along the 2 longest perpendicular axes, ranged from 3 × 6 to 8 × 8 cm. Thus entrance chambers tend to be larger than the other chambers below, which had a mean longest axis of 4.5 cm (see above). Entrance chambers were all 2–3 cm in height from floor to ceiling. In the 9 nests measured in 2010, the depth of the entrance chamber, from the ground surface to the bottom of the chamber, ranged from 3 to 8 cm, with a mean (SD) of 5.7 (1.9) cm.

All entrance chambers were a simple open polygon, but their shapes in the horizontal plane varied greatly, from oval or rectangular to heart‐shaped (Figure 2D). One of the older colonies had an entrance chamber that was a track shaped as a loop: the 2 entrance tunnels led to an oval‐shaped, 3‐cm‐wide track, about 11 cm long, around a central island of soil 5.5 cm in diameter. This loop formation is consistent with the main function of the entrance chamber as a site for encounters; while going around the loop, outgoing foragers can meet returning foragers and decide whether to leave the nest.

The entrance chamber has 2–3 tunnels that are not linked to adjacent chambers but instead go almost vertically down to the deeper nest (Figures 1, 2D, 3A). The total number of tunnels out of the entrance chamber to adjacent chambers and the deeper nest ranged from 1 to 6. The number of tunnels leading down from the entrance chamber to the deeper nest did not depend on colony age; the mean (SD) number of tunnels in young (2.7 (2.5), *n* = 7) and old (2.3 (1.3), *n* = 12) colonies leading to chambers below the entrance chamber did not differ (Mann–Whitney *U* test, *U* = 26, *p* = −0.95).

### 2.3 Changes in the Nest Entrance as Colonies Grow

A nest has 1–3 nest entrances on the mound surface, but all colonies have a single entrance chamber. In all 19 colonies whose nest entrances were excavated, all the entrance tunnels led into the same entrance chamber (illustrated in Figure 3A). All entrance tunnels were less than 2 cm wide regardless of the number of tunnels.

The number of nest entrances, and thus the number of tunnels leading to the single entrance chamber, increases with colony age. In 2024, 51 older (≥ 5 years) colonies had significantly more nest entrances than 40 younger (≤ 4 years); (mean (SD) Old: 1.7 (0.8), Young: 1.3 (0.5), *t*‐test, df 89, *t* = 2.72, *p* = 0.008). Of the 19 colonies whose nest entrances were excavated horizontally, most (9 of 12) of the mature colonies had more than one entrance with a tunnel to the entrance chamber. The oldest colony, age 23 years, had 3 entrance tunnels of which one bifurcated into two.

### 2.4 The Daily Pattern of Flow Through the Entrance Chamber

To learn about the daily pattern of movement of ants into the entrance chamber from the deeper nest, in 2010 I observed activity in the nest entrance chamber, using an Olympus Iplex LT industrial videoscope with a 0.4 mm rubber probe on a 2 m cord, with a red filter over the light to reduce the visibility of the light to the ants. Observations were made for 2–3 h on 5 days between Aug 19 and 31, 2010, during the morning activity period, of 8 active colonies, ranging in age from 1 to 23 years old. The videoscope was set up on a tripod well outside the edge of the mound away from any foraging trails. The probe was inserted from 1 to 10 cm below the nest entrance. It was sometimes possible to move the probe into the chambers adjacent to the entrance chamber. As the walls of these wide tunnels are hard, lined like the chambers with an adobe finish, the probe had no visible effect on nest structure. In older, larger colonies, there were always some ants that attempted to sting the probe of the videoscope when it was introduced, and such attempts were more prolonged, continuing for up to 20 min, later in the morning activity period, when temperatures were high and the rate of flow through the entrance tunnel was high.

Whether there were ants in the entrance chamber depended on the current foraging activity of the colony. A colony does not forage actively every day (Gordon 1991, 2013). Four colonies were observed both on days when foragers were active and when they were not. In all 4 colonies, on days when the colony was not foraging actively, the entrance tunnel and chamber were almost empty of ants, except for a few nest maintenance workers that came out of the nest to deposit loads of refuse on the nest mound.

When a colony was foraging actively, its entrance chamber and the tunnel to the outside entrance were always packed with ants moving in both directions, both toward and away from the nest entrance. Ants engaged in brief antennal contact as they moved up and down the tunnel. The ants moved on all surfaces of the entrance tunnel, without any lanes and without any apparent distinction between the top and bottom of the tunnel.

Most of the ants in the entrance tunnel were foragers moving in and out of the nest. To facilitate observation with the videoscope, in one colony on 20 Aug 2010, about 200 foragers were marked with colored paint from a Uni‐marker pen and released the same afternoon, and then observed during the morning foraging period for 30–90 min each day for the next 5 days. These marked ants moved rapidly up the entrance tunnel and out of the nest, or down the entrance tunnel carrying food to the entrance chamber. Foragers sometimes dropped their food load on the floor of the entrance tunnel, where other unmarked ants, coming up from below, picked it up and carried it down to the entrance chamber, indicating that this task is not done by foragers. A few times an unmarked ant approached a returning, marked forager with a seed, took the seed from the forager's mandibles into its own, and then took the seed down into the tunnel toward the entrance chamber. Other unmarked ants could be identified as nest maintenance workers because they came up from the entrance chamber carrying loads of soil and refuse to be deposited outside the nest.

While foragers sometimes drop their loads in the entrance tunnel, observations with the videoscope showed that food is quickly moved into the entrance chamber. To track the movement of food, for all colonies observed with the videoscope, a small pile of millet was placed along the foraging trail early in the morning; the yellow grains of millet were easy to see inside the entrance tunnel. In the first few minutes that millet was being carried into the nest, grains of millet were scattered throughout the entrance tunnel. Gradually, over about the next 10 min, more grains of millet were visible further down the entrance tunnel and fewer near the nest entrance at the top of the entrance tunnel. All millet in the tunnel was carried down into the entrance chamber within 5–10 min. For example, in one mature colony observed on 21 Aug 2010, millet was provided outside the nest at 06:55, all millet had been collected and carried into the nest by 07:15, and all of the millet brought inside had been carried down past the entrance tunnel into the entrance chamber by 07:20; similarly, in a different mature colony observed on 31 Aug 2010, all millet had disappeared from the entrance tunnel and into the entrance chamber 5 min after the pile by the foraging trail was depleted.

In previous studies conducted since 2010, we tracked from video, using image analysis, the trajectories of foragers in the entrance chambers where the top layer of soil had been excavated (Pinter‐Wollman et al. 2013; Davidson et al. 2016). Here we also saw foragers bring in seeds, drop them in the entrance chamber, and then, after antennal contact with other returning foragers, go out on another foraging trip. Other observations of individually marked foragers showed that foragers often spend only 1–2 min inside the nest between successive foraging trips (Beverly et al. 2009; Nova et al. 2022). This further supports the conclusion that foragers drop their seeds and leave the nest again without going below the entrance chamber with their seeds, because it would take more than 1–2 min for foragers to travel down to lower chambers, return to the entrance chamber, and leave the nest. When foraging activity ends, there are still seeds piled up in chambers adjacent to the entrance chamber, or in the tunnels leading to those adjacent chambers (Figure 2E). The behavior of *P. badius* is similar: seeds are transferred from chambers at the surface to seed chambers lower down (Kwapich and Tschinkel 2013; Tschinkel et al. 2015).

**FIGURE 6:**
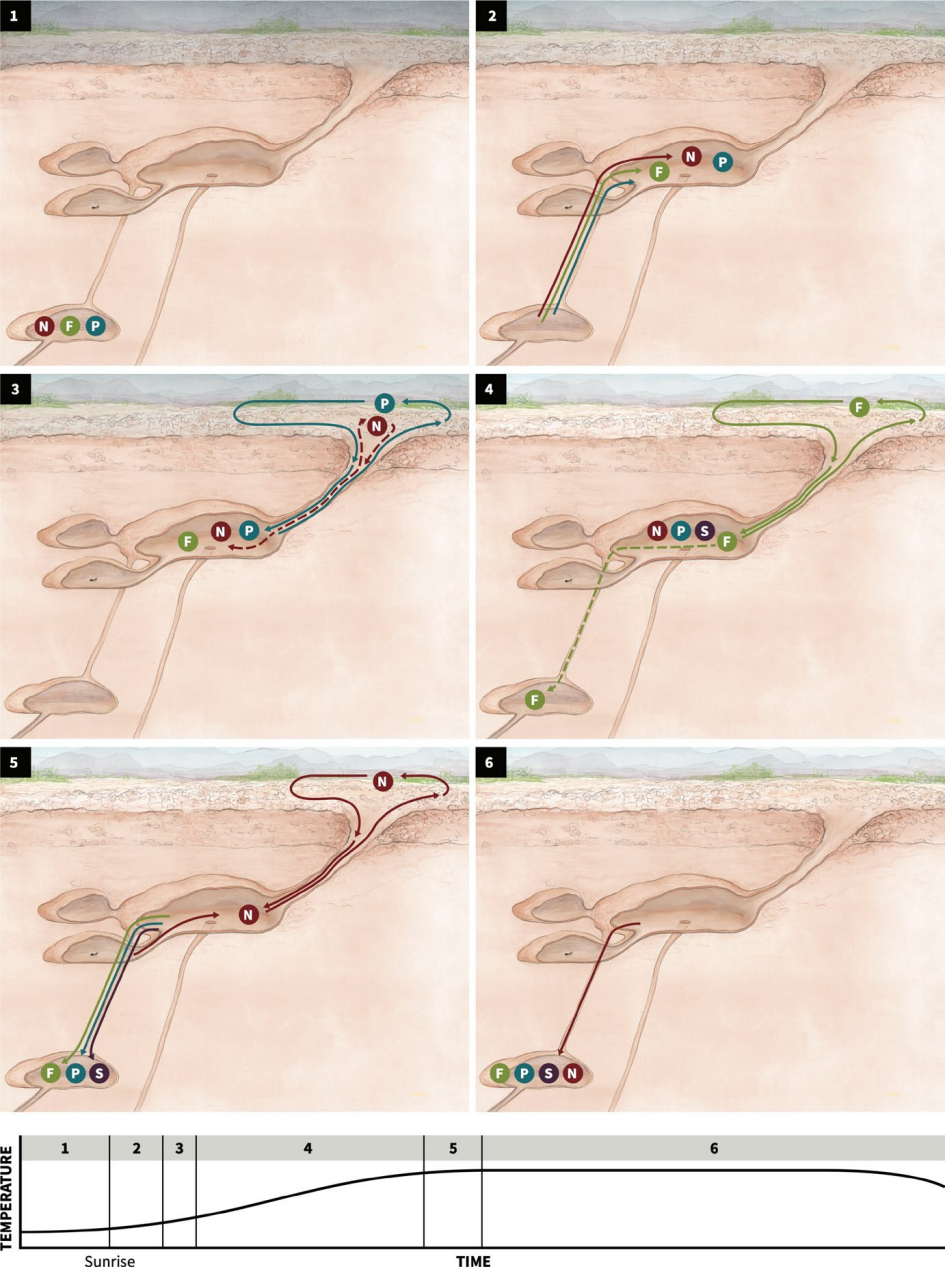
The daily pattern of the flow of ants in the entrance chamber. Illustration by Semay Johnston. The stage in the morning activity period represented by each panel is shown in the figure at the bottom. (1) When the colony is not active, exterior workers are in chambers just below the entrance chamber. (2) Patrollers (P), Nest maintenance workers (N) and Foragers (F) move to the entrance chamber. (3) Patrollers go out and return. If the nest entrance is obstructed, Nest maintenance workers clear debris. (4) The return of the patrollers initiates foraging. Foragers make many trips outside, but go down if the rate of forager return is low. Sorters (S) work inside the entrance chamber to pile food to go down to deeper chambers, and refuse to be taken out. Patrollers stay in the entrance chamber until late morning when it is hot and dry. (5) At the end of the foraging period, Foragers and Patrollers go down to the deeper chambers, and Nest maintenance workers take out refuse. (6) All exterior workers go back to the deeper chambers until the next foraging period.

Figure 6 brings together these observations and previous work to summarize the daily pattern of the flow of ants through the entrance chamber. It seems likely that the ants that work outside the nest tend to remain overnight close to the surface in chambers below the entrance chamber (Figure 6: 1), as this is common in many ant species with subterranean nests (Franks and Tofts 1994), and has been demonstrated in other *Pogonomyrmex* species (MacKay 1981; Kwapich and Tschinkel 2013; Tschinkel and Hanley 2015). Previous work in this *P. barbatus* population also indicates that exterior workers stay near the entrance chamber overnight; ants collected when working outside the nest and marked tend to come out the next day doing the same tasks (Gordon 1989; Beverly et al. 2009; Nova et al. 2022).

On a day that a colony is active outside the nest, exterior workers move up to the entrance chamber in the morning (Figure 6: 2). In the observations with a videoscope reported here, entrance chambers were empty in inactive colonies. Previous work shows that if the nest is obstructed by debris due to flooding or plugged by neighboring colonies of a competing species, *Novomessor cockerelli* (Gordon 1988), nest maintenance workers open it by removing the debris. If there is no obstruction, the patrollers emerge first soon after sunrise, travel around the foraging area, and then return to the nest (Gordon 1986) (Figure 6: 3). The safe return of the patrollers stimulates the first wave of foragers to leave the nest (Greene and Gordon 2003) (Figure 6: 4). Previous removal experiments show that if the patrollers do not return safely, more emerge, at least until late morning if humidity is low (Gordon 2002), at a rate that differs among colonies (Gordon et al. 2011). These previous studies suggest that the patrollers stay in the entrance chamber for some time after foraging begins.

The first foragers wait in the entrance chamber for the return of the patrollers and probably push upwards into the entrance tunnel, since dropping beads coated with patroller CHCs into the entrance tunnel immediately elicited the onset of foraging (Greene and Gordon 2003). Often the return of 10–30 patrollers can trigger a coherent wave of hundreds of foragers to emerge (Pagliara et al. 2018), indicating the foragers are gathered close together where they can quickly be stimulated to leave by returning patrollers.

Once foraging has begun, each forager makes many trips (Beverly et al. 2009; Nova et al. 2022) (Figure 6: 4). The outgoing foragers are stimulated to leave the nest on their next trip by the rate of encounter with foragers returning with food, depending also on the humidity conditions outside the nest (e.g., Pagliara et al. 2018). If the rate of forager return is too slow, foragers go down below the entrance chamber; removal experiments showed that foragers would wait in the entrance chamber for 3 min without any returning foragers, but tend to go down to the deeper nest if no foragers return in 8 min (Pinter‐Wollman et al. 2013). The presence of rubbish and seeds in the entrance chambers excavated at the end of the foraging period, reported here, as well as other work tracking the rate of refuse removal (Das and Gordon 2025), shows that the ants that work inside the nest sorting seeds and rubbish (indicated as ‘S’ in Figure 6: 4) are in the entrance chamber at the same time that foragers are coming in and out of the nest.

During the morning activity period, brood are brought to the chambers near the surface at the edge of the mound (Figure 1; not shown in Figure 6), presumably because brood develop faster when warm. Early in the morning, these chambers are empty, but later, on a sunny day in the summer when the ground has become warm, they are filled with larvae and pupae. If the chamber is opened from the nest surface, the workers, often including callows, rush to carry the brood further down. Workers collected from brood chambers differed from exterior workers in circadian patterns of gene expression (Ingram et al. 2016); this is consistent with the usual trend in ants that brood workers are younger than exterior workers. A similar daily pattern of brood transport occurs in lab colonies, where a nest box is heated for several hours a day. Brood are taken to this heated chamber when it is warm and then, when the heat is off, taken back to another chamber with the rest of the brood where the queen tends to stay.

Foraging continues until conditions are prohibitively hot and dry (Nova et al. 2022), at about 11 am on a clear day in the summer. At the end of the morning activity period, nest maintenance workers clear refuse out of the nest and deposit it on the midden (Figure 6: 5). Then all exterior workers move down below the entrance chamber until the next foraging period (Figure 6: 6).

### 2.5 Nest Topology

These observations show that the overall network topology of a *P. barbatus* nest is a minimum spanning tree based at the entrance chamber. At the local scale of the nest entrance and adjacent chambers, despite considerable variation in the size and shape of the chambers, the minimum spanning tree topology is maintained. There may be more than one entrance from the surface, but all entrances are linked by tunnels to a single entrance chamber. Chambers adjacent to the entrance chamber are not connected to each other.

The topology of the nest entrance and adjacent chambers has a high cost in water loss of foraging activity (Figure 6). The highly connected cluster of chambers around the nest entrance funnels ants into the entrance chamber (Pinter‐Wollman 2015a; Vaes et al. 2020), where the interactions take place that are crucial to the moment‐to‐moment regulation of foraging in response to current conditions. This structure facilitates the immediate deployment of ants outside the nest, giving higher priority to the rapid adjustment of foraging activity, to manage water loss, than to the rapid distribution of resources inside the nest. The distribution of resources inside the nest can be slow because seeds can be transported gradually and stored for many months. The dense cluster of chambers around the entrance extends into chains of chambers with low connectivity, which slows down the distribution of resources because it reduces the number of shortcuts between chambers (Pinter‐Wollman 2015b). This structure, common in many ant species (Tschinkel 2021; Miller et al. 2022; O'Fallon et al. 2023), separates the ants processing food, husking and storing seeds, and bringing food to the brood, from the ants closer to the surface bringing in seeds and bringing out the waste.

The changes in the nest entrance as colonies grow reflect the influence of nest shape on the encounters that regulate foraging activity. Older, larger colonies make more entrance tunnels, which link the nest entrance to the entrance chamber, than younger, smaller ones. More narrow tunnels, instead of one wider one as the colony grows larger, can act to encourage direct flow, with fewer ants moving sideways in the tunnel, which better reflects the true rate at which foragers return to the nest.

More entrance tunnels may also help to increase the flow of foragers leaving the nest in a large colony that makes more than 5000 foraging trips in the morning activity period (Gordon and Kulig 1996; Adler and Gordon 2003; Pagliara et al. 2018). In a laboratory study of *Myrmica rubra* , an increase in the number of entrances from a common chamber led eventually to the even distribution of workers across entrances, facilitating the flow outside the nest (Lehue and Detrain 2019). The faster ants come out of the entrance chamber, the faster more can move in, and the more likely returning foragers are to encounter an outgoing forager before they drop their food. Once a forager has dropped its food, it is less likely to stimulate foraging activity from the ants it meets; the combined odors of food and foragers stimulate foraging more than the odors of food or foragers alone (Greene et al. 2013).

The obstruction created by more than one entrance tunnel may also contribute to the speed at which foragers can leave the nest. In all nests with more than one exterior entrance, the tunnels from all entrances link to a single entrance chamber. The wall of the entrance chamber between the openings of two entrance tunnels acts as an obstacle (Figure 3A). An obstacle in the exit from an enclosed space facilitates movement out by stabilizing flow and reducing the probability of eddies, both for humans (Helbing et al. 2005) and ants (Peters et al. 2006; Burd et al. 2010).

Further work is needed to evaluate quantitatively the effect of more entrance tunnels, as a colony grows older and larger, on the rate of encounter between outgoing and returning foragers and the flow of ants in and out of the entrance chamber.

## 3 Effects of Drought on Nest Entrance Structure

### 3.1 Craters at the Nest Entrance

In 2019, after several extremely dry years (Sundaram et al. 2022), many small craters of about 10 cm deep and 20 cm wide formed in the soil surface (Figure 3B). There were at least 75 of these craters scattered around the 10‐ha study site, most often at the entrances of *P. barbatus* nests. Out of 120 active nests that were not under plants and thus exposed to the wind, 40 had a crater at the collapsed nest entrance. The craters were probably caused by wind erosion of dry clay sediment at the surface; a strong wind could lift the loose dry soil over the cluster of chambers at the nest entrance, as well as over other places where an animal had made a hole.

When the nest entrance collapsed, the sides of the crater exposed some chambers below the nest entrance. The colony used at least one of the open, newly exposed chambers as the nest entrance for foraging, and did not attempt to cover the open chambers. To assess the rate of repair of open chambers, 6 nests with collapsed nest entrances were monitored, using photos of the nest of each colony on 5–8 of the 12 days between 17 and 28 Aug 2019. The number of chambers opened by the collapse of the area around the entrance ranged from 2 to 8. None of the colonies repaired the exposed chambers on the edge of the collapsed area in the course of the 12 days of observation; all 6 colonies had the same number of opened chambers on the last day of observation as on the first. One colony closed a chamber during the 10 days but opened it again by the last day. In the following year, 2020, all 6 colonies had fully repaired their nests; all had intact nest mounds with one nest entrance and no open chambers.

The collapse of nest entrances did not have a significant effect on colony survival. Of 35 nests collapsed in August 2019, 5 (16.6%) had died by August 2020; of 94 not collapsed in Aug 2019, a similar proportion, 11 (13.3%), had died by Aug 2020 (Fisher's exact test, ns, p 0.76). Survival is associated with colony age (Sundaram et al. 2022), and the colonies that died in both groups spanned a similar range of ages.

In some of the colonies with collapsed nest entrances, there were 2 entrances out of newly opened chambers, one on either side of the crater, with a foraging trail from each. In intact nests, if there are two entrances, they lead to the same entrance chamber. This raised the question whether colonies could regulate foraging from two separate chambers. Because the nest entrance is a hub linked to diverging chains of chambers, it is unlikely that two distant chambers separated by a crater happen to be connected to a third chamber further down.

In one colony with foraging trails from two exposed chambers created by a crater, a removal experiment indicated that foraging was regulated from two distinct chambers. In intact nests, removing returning foragers, decreasing the rate at which foragers return to meet outgoing foragers, leads to a decrease in the rate at which outgoing foragers leave the nest (e.g., Gordon et al. 2011; Prabhakar et al. 2012). Removing returning foragers from the trail to one entrance did not influence the foraging rate on the other trail, in 2 trials with one colony on Aug 19 and 21 2013. Methods were as in previous work (e.g., Prabhakar et al. 2012): foraging rates were measured on both trails and foragers were removed for 3 min on one of the trails, while the other trail was left undisturbed. Results from one trial are shown in Appendix S1; results from the other were similar.

In colonies with collapsed entrances and two foraging trails into two exposed chambers, there was also a trail of ants inside the crater between the two exposed chambers. These ants were not carrying seeds, suggesting that they were outgoing foragers. In intact nests, a forager tends to use the same trail day after day (Gordon 1991) and to search at the same site along that trail in successive trips within a day (Beverly et al. 2009). Apparently, the separation of ants into different entrance chambers disrupted whatever cues normally direct a forager onto the same trail it used on the previous trip.

I excavated the entrances of two nests with collapsed nest entrances. Both colonies were using only one of the exposed chambers as the nest entrance. In both colonies, the open chamber currently used as a nest entrance led inside to a tunnel and then another chamber, smaller than a normal entrance chamber, that was being used as the entrance chamber.

### 3.2 Crumbling of the Nest Surface

In 2023, temperatures were unusually high and there was no rain at all at the site in July or August. The surface of at least 40 nest mounds cracked open, often exposing the entrance chamber at the center of the mound, the brood chambers at the outer edges of the mound, or both (Figure 3D). Most of the damaged nests were in the region of the site where the previous year, mudflow after heavy rainfall covered many nests (Figure 3C). When the ceiling of the brood chambers at the outer edges of the mound crumbled, ants were trapped outside, as there are few tunnels to any other chambers from these peripheral chambers (Figure 1). Since the ants that care for the brood tend to be younger, these ants had probably never been outside the nest.

In 22 of the 40 nests, there were more than 3 exposed chambers and piles of dead or dying ants on the nest surface. Pouring water on the nest surface brought more ants to the surface who were then exposed to the heat. I excavated one nest with a severely crumbled mound surface near but not on the study site. Inside the nest, beyond an intact entrance chamber, there was just one large, open, dusty space, though some of the peripheral chambers near the surface remained intact. The usual dense mass of small chambers around the nest entrance was gone.

### 3.3 Resilience to Climate Change

The minimum spanning tree structure provides some resilience to disturbance, because the brood, queen, and stored food are protected from flooding and other intrusions from outside the nest when at the ends of chains of chambers extending from the cluster around the entrance. However, a minimum spanning tree structure is susceptible to failure at its root or hub, in this case the entrance chamber.

Humidity inside the nest normally remains high throughout the day despite the rapid drop in humidity outside (Pagliara et al. 2018). Humidity is kept high throughout the nest in small chambers, with surfaces sealed by an adobe finish, filled with respiring ants. The entrance chamber, where foragers recover before their next trip outside, may be the most humid place in the nest because it is in the upper clay layer of the soil.

When soil is not dry, wind erosion is not severe, and the clay adobe surfaces do not shrink, a nest can remain intact without any further maintenance for several years after a colony dies. Sometimes a neighboring colony moves into a nest whose colony has died, or a neighbor goes into the abandoned nest and takes the seeds. In 2022, a neighboring colony took seeds from a nest that had been abandoned for 3 years, indicating that the seed chambers in the abandoned nest were still intact.

As drought and rising temperatures have led to drier soils that are more susceptible to collapse and erosion, the risk of losing the humid entrance chamber has increased. A colony cannot forage without engaging in the encounters that initiate and regulate foraging. When entrance chambers were destroyed by the collapse of entrances from wind erosion in 2019, another chamber was used as the entrance chamber. The adobe layer around all surfaces of each chamber, built by plastering the clay sediment when moist, provides structural support. The chambers near the surface built in the clay layer, exposed to the sun, are especially vulnerable to shrink and crack when temperatures are high, as in 2023 in colonies with nests covered by clay silt after flooding in 2022.

The effects on soil of climate change, especially drought and high temperatures, can diminish a colony's capacity to manage water loss and so to survive drought in two ways: first, by directly exposing the entrance chamber to dry air and second, by disrupting the encounters in the entrance chamber that regulate forager exposure to low humidity outside the nest.

Colonies differ in how they regulate foraging activity to manage water loss; some sacrifice food intake to conserve water by reducing activity on the driest days, while others continue to forage (Gordon et al. 2023). If colonies also differ in how well they are able to maintain the nest in increasingly dry conditions, this may further increase variation among colonies in foraging activity. Differences among colonies in how they build and maintain nests lead to differences in their resilience to the effects of drought on soil, and may be crucial in adaptation to climate change.

## Author Contributions

**Deborah M. Gordon:** conceptualization (equal), data curation (equal), formal analysis (equal), funding acquisition (equal), investigation (equal), methodology (equal), project administration (equal).

## Conflicts of Interest

The author declares no conflicts of interest.

## Supporting information


**Appendix S1:** ece372122‐sup‐0001‐AppendixS1.zip.

## Data Availability

All data are in the manuscript.
