# Supplementary material for: Nest Entrance Architecture and the Regulation of Foraging Activity in Desert Harvester Ants
Source: Ecol Evol. 2025 Sep 6;15(9):e72122. doi: 10.1002/ece3.72122 (PMC12413562; doi:10.1002/ece3.72122)
Supplement: Supplementary file 1 — Appendix S1: ece372122‐sup‐0001‐AppendixS1.zip. [file ECE3-15-e72122-s001.zip › Appendix Figure 1.docx]

Appendix Figure 1. Removal experiment in a colony with 2 nest entrances due to nest entrance collapse. Red, rate of returning foragers; Blue; rate of outgoing foragers. A. Trail from which foragers were removed during the interval indicated with a black bar. B. Trail from which no foragers were removed.
