# Supplementary figures and images for: Nest Entrance Architecture and the Regulation of Foraging Activity in Desert Harvester Ants
Source: Ecol Evol. 2025 Sep 6;15(9):e72122. doi: 10.1002/ece3.72122 (PMC12413562; doi:10.1002/ece3.72122)

Foragers per 10 sec

A

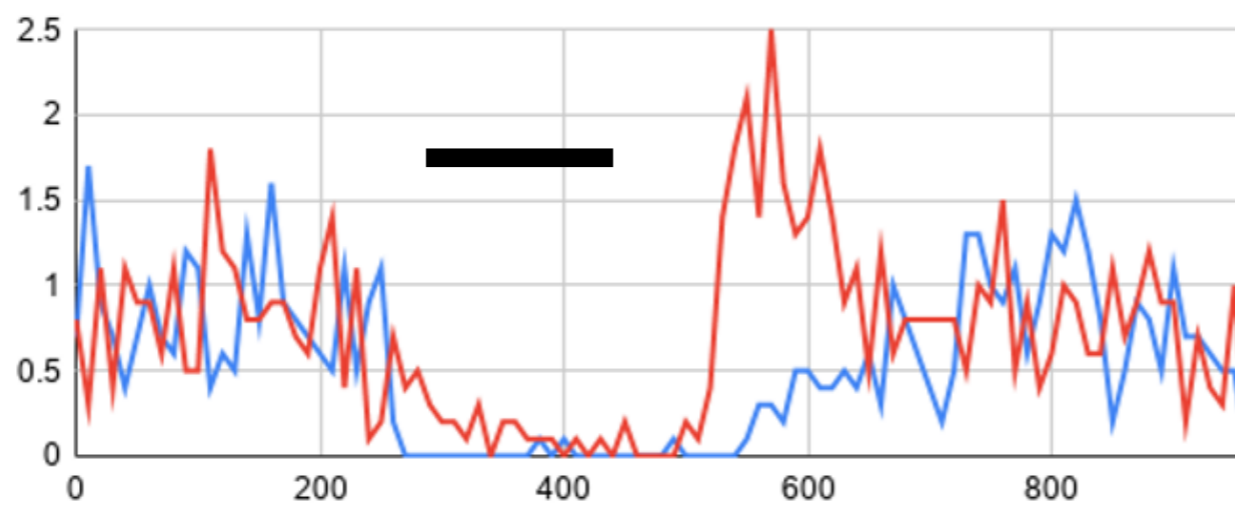

B

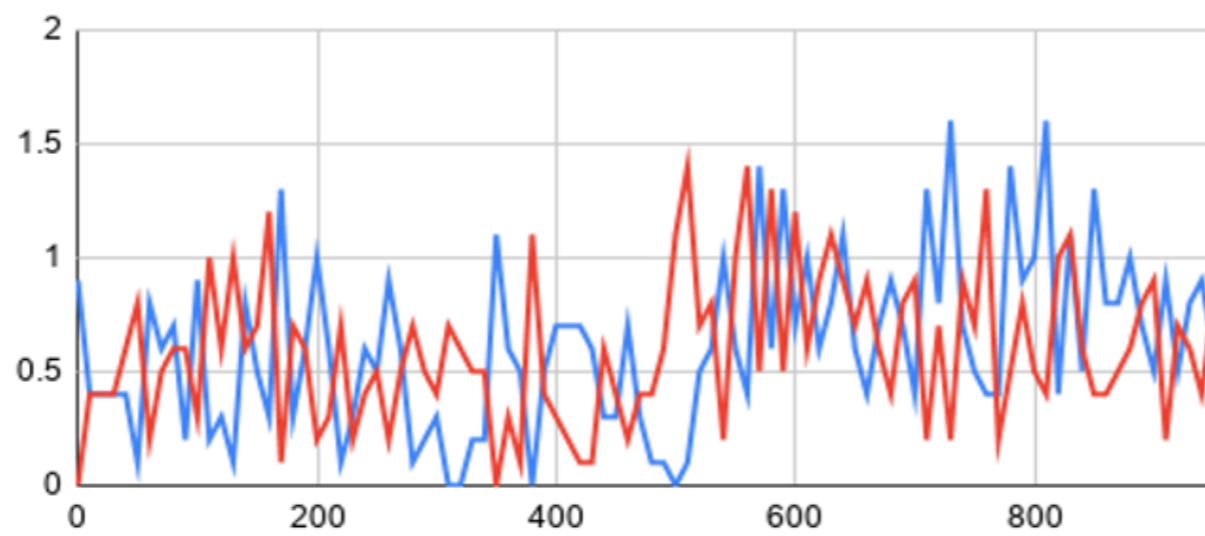

Seconds

Supplement: Supplementary file 1 — Appendix S1: ece372122‐sup‐0001‐AppendixS1.zip. [file ECE3-15-e72122-s001.zip › ECE3_72122_f1_Appendix fig 1 pdf.pdf]
